# The Role of Serial Ultrasounds in Diagnosing Suspected Deep Venous Thrombosis

**DOI:** 10.7759/cureus.4337

**Published:** 2019-03-28

**Authors:** Rolando Cabrera, Niharika Chimalakonda, Javier Rosario, Latha Ganti

**Affiliations:** 1 Emergency Medicine, University of Central Florida College of Medicine / Hospital Corporation of America Graduate Medical Education (HCA GME) Consortium, Orlando, USA; 2 Internal Medicine, University of Central Florida College of Medicine, Orlando, USA; 3 Emergency Medicine, University of Central Florida College of Medicine, Orlando, USA; 4 Emergency Medicine, University of Central Florida College of Medicine / Hospital Corporation of America Graduate Medical Education (HCA GME) Consortium, Kissimmee, USA

**Keywords:** dvt, vte, embolism, doppler, ultrasound, vascular, pe, deep venous thrombosis, venous thromboembolism, blood clot

## Abstract

Venous thromboembolisms (VTE), which include deep venous thrombosis (DVT) and pulmonary embolism (PE), are a major cause of morbidity and mortality in the United States and globally. This often underdiagnosed medical condition has many known risk factors including pregnancy, malignancy, immobility, exogenous estrogen use, and hereditary factors. A significant portion of emergency department visits involves ruling out these diseases.

This case presents a woman with unilateral leg pain and swelling who initially had a negative emergency room workup including a negative lower extremity Doppler study. Upon a repeat visit, she was found to have extensive deep venous thrombosis and was diagnosed with B-cell lymphoma. This case highlights the importance of having patients return for repeat imaging if Dopplers are negative in the initial encounter.

## Introduction

For patients with suspected deep venous thrombosis who have negative venous Doppler studies on their initial encounter, it is the standard of care to encourage return to the emergency department (ED) or their primary physician for repeat Doppler studies in one week. Approximately 2% of patients who return for their repeat studies are found to have proximal deep venous thrombosis one week later [1]. The standard lower extremity ultrasound typically goes only as distal as the popliteal vessels. It is estimated that about 5% of patients with lower extremity Dopplers that are negative actually do have a more distal clot in the calf [2].

Approximately 300,000-600,000 individuals in the United States each year are affected by this condition [3]. Roughly 60,000-100,000 of those people die as a result of their deep venous thrombosis (DVT)/ pulmonary embolism (PE). For an alarming number of people (25%), sudden death is the first symptoms of their PE [4]. The gold standard for diagnosis of DVT and PE is venography and pulmonary angiography, respectively. Lower extremity Dopplers and computed tomography (CT) angiogram of the pulmonary arteries are the standard tests to obtain in the ED due to them being readily available and less invasive.

## Case presentation

A 72-year-old woman with a medical history of coronary artery disease, ischemic cardiomyopathy, carotid artery stenosis, renal artery stenosis, chronic kidney disease, hypertension, and hyperlipidemia presented to the ED with two months of right lower extremity pain and swelling. She stated that both symptoms had worsened to the point where she was having difficulty ambulating. The patient also related new symptoms of tingling in her right foot that had started today. She revealed that she had a lower extremity ultrasound done 11 days ago with the onset of these symptoms.

**Figure 1:**
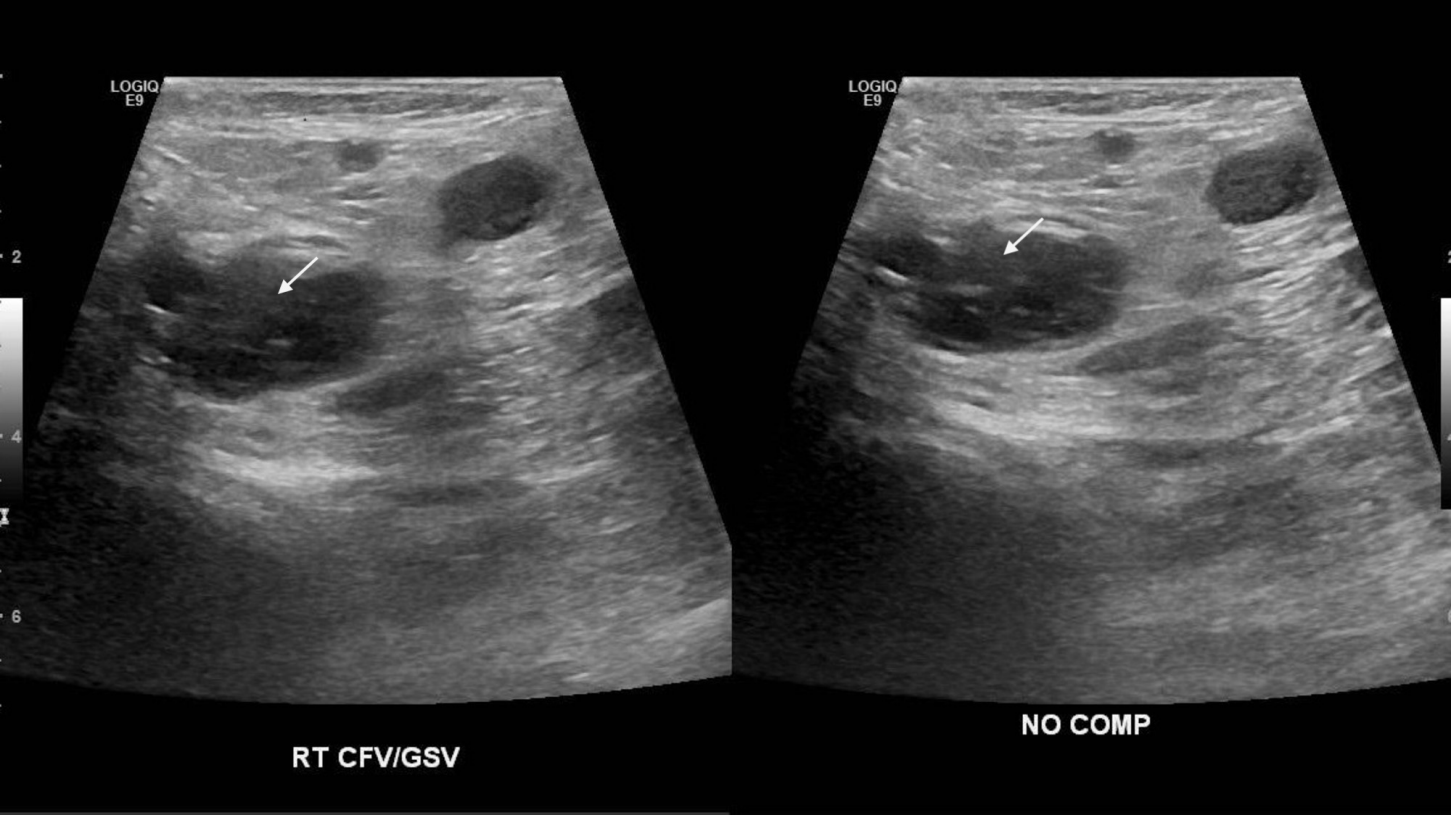
Right Common Femoral Vein (noncompressible) Looking at the left and right pictures, one notes that the right common femoral vein size is approximately the same with and without compression (arrows), which is indicative of a deep venous thrombosis (the DVT prevents compression of the vein, which would otherwise appear flat with compression).

**Figure 2:**
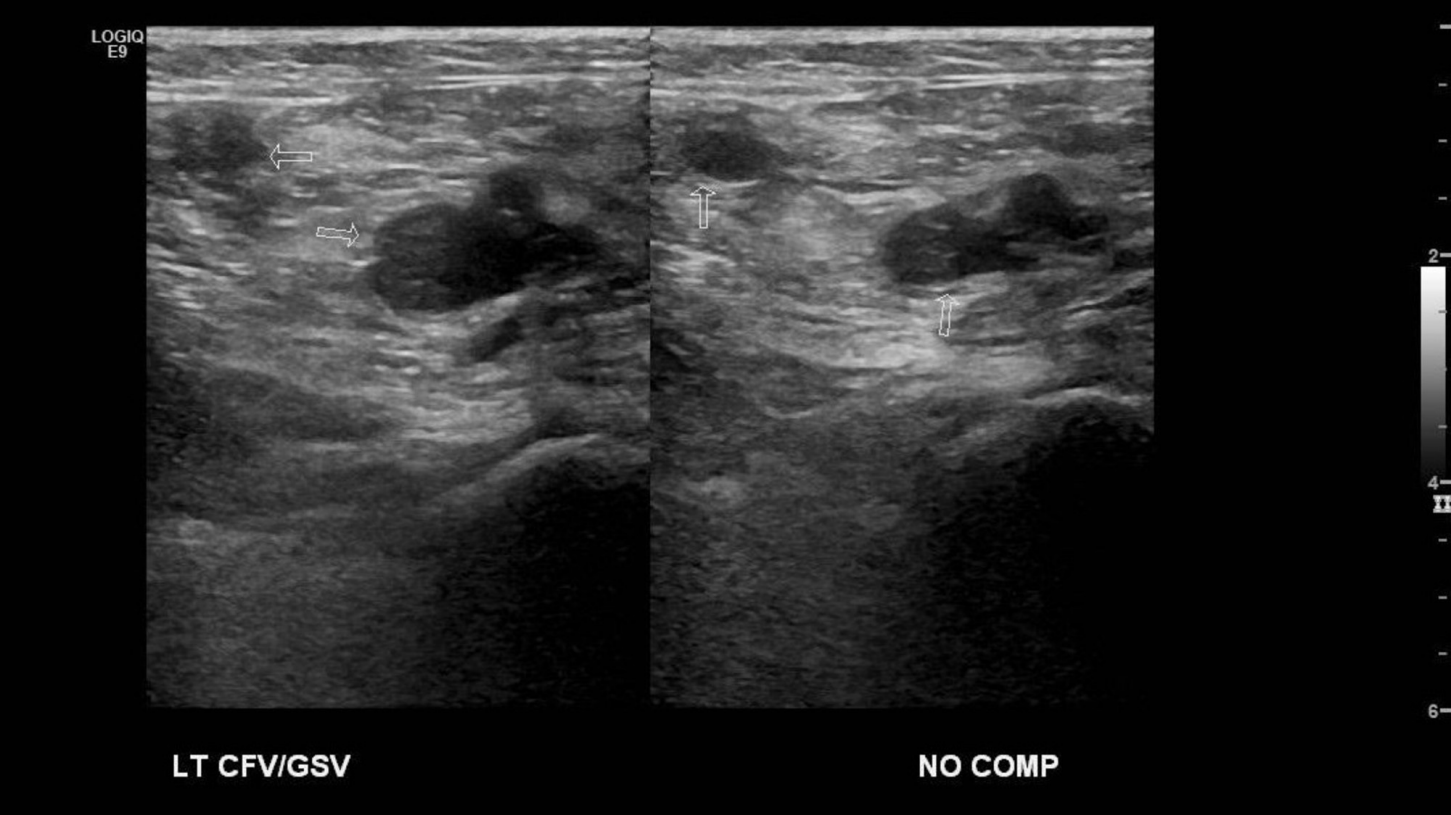
Left Common Femoral Vein

Her initial vital signs were within normal limits. Her physical exam was remarkable for an enlarged, cold, discolored right lower extremity with calf tenderness and non-palpable or Dopplerable pulses in the foot. The vascular surgeon was consulted and recommended the patient be given a heparin bolus and placed on a heparin drip with a lower extremity venogram ordered for the morning. Meanwhile, a bilateral lower extremity ultrasound was done showing right-sided DVTs (Figure 1-2). CT of the abdomen and pelvis was done without contrast due to the patient's chronic kidney disease. A ventilation perfusion scan (V/Q) was ordered and care was transferred to the inpatient team.

**Figure 3:**
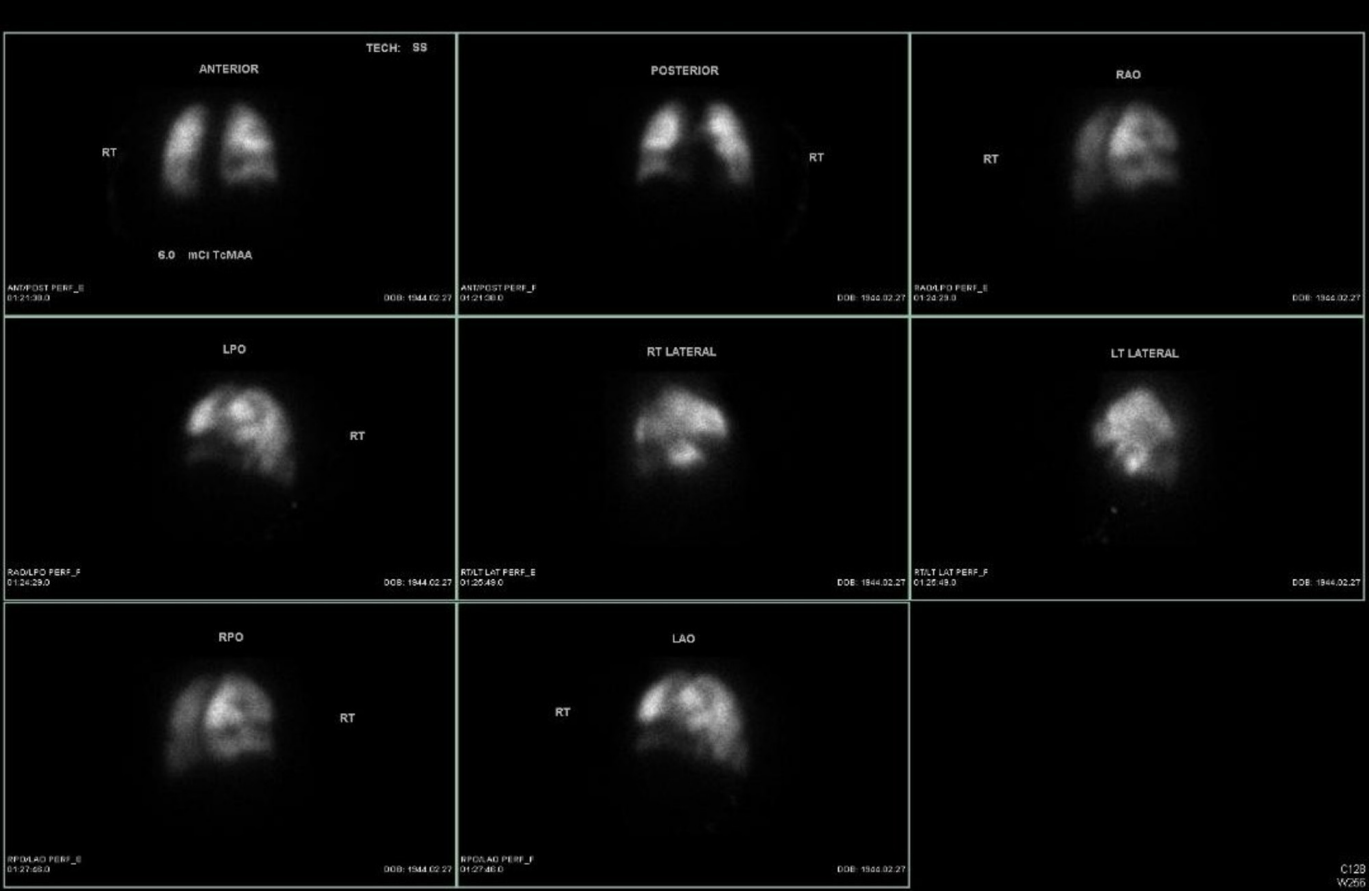
Ventilation-perfusion Scan Showing Probability of a Pulmonary Embolism

After the patient was admitted to the medical ward with a heparin drip in place, she underwent a ventilation-perfusion (V/Q) scan which showed a high probability of a PE (Figure 3). CT abdomen without contrast on admission showed only bilateral hydronephrosis secondary to enlarged para-aortic lymph nodes.

During her hospitalization, she was maintained on the heparin drip for the management of her right-sided DVT. However, despite the heparin drip and maintenance of an appropriate partial thromboplastin time, her right leg pain and swelling did not improve. Vascular surgery took her to undergo a catheter-directed thrombolysis and angioplasty of right leg, after which her symptoms improved.

She underwent a left para-aortic lymph node biopsy and a subsequent inferior vena cava (IVC) filter placement. However, her hospital course was complicated and lengthened, as she required three lymph node biopsies due to insufficient and necrotic tissue on the prior biopsies. Her final biopsy was done on her right inguinal region and showed grade three follicular B cell lymphoma.

Her situation was further complicated when she developed acute right inguinal swelling and pain a few days after her biopsy while she was on the heparin. Concern for a possible arterial bleed led to a CT scan of the extremity which revealed a hyperdense fluid collection with air fluid levels within the anteromedial right thigh, measuring 6.4 x 13.2 x 14 cm without contrast extravasation. The patient was started on vancomycin and piperacillin-tazobactam for presumed infected hematoma and underwent urgent incision and drainage. Her antibiotics were stopped after two days when cultures from the site were negative for any organisms.

The patient ultimately underwent a bone marrow biopsy that showed CD-10 and CD-20 positive lymphocytes and minimal lymphocytic infiltration into the marrow. She was started chemotherapy and was transitioned to warfarin from low molecular weight heparin until her international normalized ratio (INR) was therapeutic and she was discharged home.

## Discussion

Physical examination clues for DVT include a swollen extremity that is tender to palpation. Erythema of the skin may or may not be present but is a feature of venous engorgement. Venous ultrasound is the standard imaging test for patients suspected of having acute DVT, and the Society of Radiologists in Ultrasound recommends a comprehensive duplex ultrasound protocol from thigh to ankle with Doppler at selected sites rather than a limited or complete compression-only examination [5]. 

The mainstay of lower extremity DVT treatment is anticoagulation. Anticoagulation is imperative to reduce clot propagation and minimize the morbidity and mortality associated with this relatively common disease. Patients can be prescribed low molecular weight heparins (injected subcutaneously), or an oral anticoagulant (Table 1). If there is a concern for recurrent DVTs, as in this case, an IVC filter is placed to prevent subsequent DVTs from traveling to the lungs (pulmonary embolism).

**Table 1:** Anticoagulant Options for the Treatment of DVTs DVT - deep venous thrombosis

Class	Drug names	Route	Notes
Standard unfractionated heparin	Heparin	Intravenous or subcutaneous	Usually requires hospital stay for monitoring and dose adjustment
Low molecular weight heparins	Dalteparin, enoxaparin, tinzaparin	Subcutaneous	Hospital stay not required
Vitamin K antagonist	Warfarin	Oral	Requires frequent outpatient monitoring including blood draws to ensure optimum anticoagulation level
Direct thrombin inhibitor	Dabigatran	Oral	Frequent monitoring not required, but kidney function must be monitored periodically
Direct factor Xa inhibitor	Rivaroxaban, edoxaban, apixaban	Oral	Frequent monitoring not required, but kidney function must be monitored periodically

Most hospitals do not offer whole leg scans that include the venous system distal to the knee. This is due to the fact that they are not only more time consuming, but they are also technically more difficult. Whole leg scans that do not show a DVT do not typically need repeat imaging to rule out DVT. When proximal leg Doppler scans are negative, repeat Doppler studies are only positive in about 2% of patients. Despite this, having patients follow-up in one week for a repeat venous Doppler after an initial negative study continues to be the standard of care in the emergency department. The continuation of this practice emphasizes the importance of successful diagnosis and the initiation of treatment for venous thromboembolisms.

## Conclusions

This case report presents a scenario in which a patient with signs and symptoms of a DVT had a normal initial workup and was subsequently found to have a DVT upon a follow-up visit. Primary care providers and emergency physicians should have a high level of suspicion for DVT in patients who have continued symptoms.
